# Supplementary material for: A landscape evaluation of caffeine citrate availability and use in newborn care across five low- and middle-income countries
Source: PLOS Glob Public Health. 2024 Jul 29;4(7):e0002486. doi: 10.1371/journal.pgph.0002486 (PMC11285928; doi:10.1371/journal.pgph.0002486)
Supplement: S3 Data — (DOCX) [file pgph.0002486.s004.docx]

**Supplement - Forecast data Inputs and Assumption**

**Notes**

Ethiopia: CC is not currently available in the public sector in Ethiopia, but there is a belief that a small amount of penetration is achievable in the base case by 2026. India: India is the only focus country where CC is currently the predominant AOP drug in the public sector. It is also the only focus country with local manufacturing of CC. All Countries: Across all focus countries, moderate and high scenarios assume the same trajectory (to a dominant share position by 2026) for CC % share. The difference between the moderate and high scenarios is in the assumed approach to prophylaxis provision.

| **Estimated CC % of AOP drug provision in the public sector** | | | | | | | | | | |
| --- | --- | --- | --- | --- | --- | --- | --- | --- | --- | --- |
|  | **Ethiopia** | | **India** | | **Kenya** | | **Nigeria** | | **South Africa** | |
|  | ***2021*** | ***2026*** | ***2021*** | ***2026*** | ***2021*** | ***2026*** | ***2021*** | ***2026*** | ***2021*** | ***2026*** |
| **High case** | 0% | 70% | 85% | 95% | 23% | 85% | 5% | 75% | 20% | 85% |
| **Moderate case** | 0% | 70% | 85% | 95% | 23% | 85% | 5% | 75% | 20% | 85% |
| **Base case** | 0% | 5% | 85% | 85% | 23% | 23% | 5% | 5% | 20% | 20% |

**All Countries**

**Ethiopia**

**National births**

| **Indicator** | | **Datapoint** |
| --- | --- | --- |
| **Total births (#k)** | *In 2021* | 3,663 |
|  | *In 2026* | 3,889 |
| **Annual growth in # births (2021-26)** | | 1.2% |
| **Sectoral split of birth location** | *Public* | 72% |
|  | *Private* | 5% |
|  | *Non-facility (home/community)* | 23% |
| **Split of births in public sector** | *Lower-level facilities** | 70% |
|  | *Higher-level facilities** | 30% |
| **Preterm/full term split of births** | *Preterm* | 11% |
|  | *Full term* | 89% |
| **Split of preterm births by gestational age sub-category** | *Extremely preterm (<28 wks)* | 9% |
|  | *Very preterm (28-32 wks)* | 21% |
|  | *Moderate/late preterm (32-37 wks)* | 70% |

**Referrals**

| **Indicator** | | **Datapoint** |
| --- | --- | --- |
| **Referrals – private sector to public sector:** | | |
| **% of preterm cases referred** | *Extremely preterm* | 10% |
|  | *Very preterm* | 10% |
|  | *Moderate/late preterm* | 10% |
| % attempted referrals unsuccessful** | | 50% |
| **Referrals – non-facility sector to public sector:** | | |
| **% of preterm cases referred** | *Extremely preterm* | 100% |
|  | *Very preterm* | 100% |
|  | *Moderate/late preterm* | 50% |
| % attempted referrals unsuccessful** | | 50% |
| **Referrals – lower-level public to higher-level public facilities:** | | |
| **% of preterm cases referred** | *Extremely preterm* | 100% |
|  | *Very preterm* | 90% |
|  | *Moderate/late preterm* | 70% |
| % attempted referrals unsuccessful** | | 50% |

**AOP Incidence**

| **Indicator** | | **Datapoint** |
| --- | --- | --- |
| **% preterm infants developing AOP** | *Extremely preterm* | 95% |
|  | *Very preterm* | 80% |
|  | *Moderate/ late preterm* | 25% |

**Treatment/prophylaxis provision assumptions (public sector facilities with ability to treat)**

| **Indicator** | | **Base and Moderate cases** | | **High case** | |
| --- | --- | --- | --- | --- | --- |
|  |  | *2021* | *2026* | *2021* | *2026* |
| **AOP cases – provision of drug treatment** | | | |  | |
| **% of AOP (preterm) cases requiring drug treatment** | *Extremely preterm* | 100% | 100% | 100% | 100% |
|  | *Very preterm* | 100% | 100% | 100% | 100% |
|  | *Moderate/late preterm* | 100% | 100% | 100% | 100% |
| **Non-AOP cases – provision of drug prophylaxis** | | | |  | |
| **% of non-AOP (preterm) cases requiring drug prophylaxis** | *Extremely preterm* | 100% | 100% | 100% | 100% |
|  | *Very preterm* | 100% | 100% | 100% | 100% |
|  | *Moderate/late preterm* | 0% | 0% | 0% | 100% |
| **At-facility provision constraint** | | | |  | |
| **% preterm cases requiring AOP drugs but not able to receive them (due to e.g. stock availability, bed/equipment capacity, HW capacity/skill)** | | 10% | | | |

**India**

**High-level birth 5 states in India (Delhi, Bihar, Uttar Pradesh, Telangana and Madhya Pradesh)**

| **Indicator** | | **Datapoint** |
| --- | --- | --- |
| **Total births (#k)** | *In 2021* | 10,954 |
|  | *In 2026* | 10, 356 |
| **Annual growth in # births (2021-26)** | | -1.1% |
| **Sectoral split of birth location** | *Public* | 59% |
|  | *Private* | 36% |
|  | *Non-facility (home/community)* | 5% |
| **Split of births in public sector** | *Lower-level facilities** | 60% |
|  | *Higher-level facilities** | 40% |
| **Preterm/full term split of births** | *Preterm* | 12% |
|  | *Full term* | 88% |
| **Split of preterm births by gestational age sub-category** | *Extremely preterm (<28 wks)* | 12% |
|  | *Very preterm (28-32 wks)* | 30% |
|  | *Moderate/late preterm (32-37 wks)* | 58% |

**Referrals**

| **Indicator** | | **Datapoint** |
| --- | --- | --- |
| **Referrals – private sector to public sector:** | | |
| **% of preterm cases referred** | *Extremely preterm* | 10% |
|  | *Very preterm* | 10% |
|  | *Moderate/late preterm* | 10% |
| % attempted referrals unsuccessful** | | 20% |
| **Referrals – non-facility sector to public sector:** | | |
| **% of preterm cases referred** | *Extremely preterm* | 90% |
|  | *Very preterm* | 90% |
|  | *Moderate/late preterm* | 20% |
| % attempted referrals unsuccessful** | | 35% |
| **Referrals – lower-level public to higher-level public facilities:** | | |
| **% of preterm cases referred** | *Extremely preterm* | 80% |
|  | *Very preterm* | 70% |
|  | *Moderate/late preterm* | 50% |
| % attempted referrals unsuccessful** | | 20% |

**AOP Incidence**

| **Indicator** | | **Datapoint** |
| --- | --- | --- |
| **% preterm infants developing AOP** | *Extremely preterm* | 77% |
|  | *Very preterm* | 58% |
|  | *Moderate/ late preterm* | 19% |

**Treatment/prophylaxis provision assumptions (public sector facilities with ability to treat)**

| **Indicator** | | **Base and Moderate cases** | | **High case** | |
| --- | --- | --- | --- | --- | --- |
|  |  | *2021* | *2026* | *2021* | *2026* |
| **AOP cases – provision of drug treatment** | | | | | |
| **% of AOP (preterm) cases requiring drug treatment** | *Extremely preterm* | 95% | 95% | 95% | 100% |
|  | *Very preterm* | 90% | 90% | 90% | 100% |
|  | *Moderate/late preterm* | 85% | 85% | 85% | 100% |
| **Non-AOP cases – provision of drug prophylaxis** | | | | | |
| **% of non-AOP (preterm) cases requiring drug prophylaxis** | *Extremely preterm* | 100% | 100% | 100% | 100% |
|  | *Very preterm* | 50% | 50% | 50% | 100% |
|  | *Moderate/late preterm* | 10% | 10% | 10% | 100% |
| **At-facility provision constraint** | | | | | |
| **% preterm cases requiring AOP drugs but not able to receive them (due to e.g. stock availability, bed/equipment capacity, HW capacity/skill)** | | 7.5% | | | |

**National births**

| **Indicator** | | **Datapoint** |
| --- | --- | --- |
| **Total births (#k)** | *In 2021* | 1,518 |
|  | *In 2026* | 1,576 |
| **Annual growth in # births (2021-26)** | | 0.8% |
| **Sectoral split of birth location** | *Public* | 62% |
|  | *Private* | 13% |
|  | *Non-facility (home/community)* | 25% |
| **Split of births in public sector** | *Lower-level facilities** | 80% |
|  | *Higher-level facilities** | 20% |
| **Preterm/full term split of births** | *Preterm* | 18% |
|  | *Full term* | 82% |
| **Split of preterm births by gestational age sub-category** | *Extremely preterm (<28 wks)* | 3% |
|  | *Very preterm (28-32 wks)* | 16% |
|  | *Moderate/late preterm (32-37 wks)* | 81% |

**Referrals**

| **Indicator** | | **Datapoint** |
| --- | --- | --- |
| **Referrals – private sector to public sector:** | | |
| **% of preterm cases referred** | *Extremely preterm* | 10% |
|  | *Very preterm* | 10% |
|  | *Moderate/late preterm* | 10% |
| % attempted referrals unsuccessful** | | 20% |
| **Referrals – non-facility sector to public sector:** | | |
| **% of preterm cases referred** | *Extremely preterm* | 100% |
|  | *Very preterm* | 100% |
|  | *Moderate/late preterm* | 100% |
| % attempted referrals unsuccessful** | | 50% |
| **Referrals – lower-level public to higher-level public facilities:** | | |
| **% of preterm cases referred** | *Extremely preterm* | 100% |
|  | *Very preterm* | 100% |
|  | *Moderate/late preterm* | 85% |
| % attempted referrals unsuccessful** | | 50% |

**AOP Incidence**

| **Indicator** | | **Datapoint** |
| --- | --- | --- |
| **% preterm infants developing AOP** | *Extremely preterm* | 100% |
|  | *Very preterm* | 85% |
|  | *Moderate/ late preterm* | 20% |

**Treatment/prophylaxis provision assumptions (public sector facilities with ability to treat)**

| **Indicator** | | **Base and Moderate cases** | | **High case** | |
| --- | --- | --- | --- | --- | --- |
|  |  | *2021* | *2026* | *2021* | *2026* |
| **AOP cases – provision of drug treatment** | | | |  | |
| **% of AOP (preterm) cases requiring drug treatment** | *Extremely preterm* | 100% | 100% | 100% | 100% |
|  | *Very preterm* | 100% | 100% | 100% | 100% |
|  | *Moderate/late preterm* | 100% | 100% | 100% | 100% |
| **Non-AOP cases – provision of drug prophylaxis** | | | |  | |
| **% of non-AOP (preterm) cases requiring drug prophylaxis** | *Extremely preterm* | 100% | 100% | 100% | 100% |
|  | *Very preterm* | 100% | 100% | 100% | 100% |
|  | *Moderate/late preterm* | 35% | 35% | 35% | 100% |
| **At-facility provision constraint** | | | |  | |
| **% preterm cases requiring AOP drugs but not able to receive them (due to e.g. stock availability, bed/equipment capacity, HW capacity/skill)** | | 35% | | | |

**Nigeria**

**National births**

| **Indicator** | | **Datapoint** |
| --- | --- | --- |
| **Total births (#k)** | *In 2021* | 7,743 |
|  | *In 2026* | 8,288 |
| **Annual growth in # births (2021-26)** | | 1.4% |
| **Sectoral split of birth location** | *Public* | 70% |
|  | *Private* | 25% |
|  | *Non-facility (home/community)* | 5% |
| **Split of births in public sector** | *Lower-level facilities** | 30% |
|  | *Higher-level facilities** | 70% |
| **Preterm/full term split of births** | *Preterm* | 15% |
|  | *Full term* | 85% |
| **Split of preterm births by gestational age sub-category** | *Extremely preterm (<28 wks)* | 21% |
|  | *Very preterm (28-32 wks)* | 34% |
|  | *Moderate/late preterm (32-37 wks)* | 45% |

**Referrals**

| **Indicator** | | **Datapoint** |
| --- | --- | --- |
| **Referrals – private sector to public sector:** | | |
| **% of preterm cases referred** | *Extremely preterm* | 50% |
|  | *Very preterm* | 30% |
|  | *Moderate/late preterm* | 10% |
| % attempted referrals unsuccessful** | | 50% |
| **Referrals – non-facility sector to public sector:** | | |
| **% of preterm cases referred** | *Extremely preterm* | 100% |
|  | *Very preterm* | 80% |
|  | *Moderate/late preterm* | 50% |
| % attempted referrals unsuccessful** | | 50% |
| **Referrals – lower-level public to higher-level public facilities:** | | |
| **% of preterm cases referred** | *Extremely preterm* | 100% |
|  | *Very preterm* | 80% |
|  | *Moderate/late preterm* | 50% |
| % attempted referrals unsuccessful** | | 50% |

**AOP Incidence**

| **Indicator** | | **Datapoint** |
| --- | --- | --- |
| **% preterm infants developing AOP** | *Extremely preterm* | 95% |
|  | *Very preterm* | 80% |
|  | *Moderate/ late preterm* | 25% |

**Treatment/prophylaxis provision assumptions (public sector facilities with ability to treat)**

| **Indicator** | | **Base and Moderate cases** | | **High case** | |
| --- | --- | --- | --- | --- | --- |
|  |  | *2021* | *2026* | *2021* | *2026* |
| **AOP cases – provision of drug treatment** | | | |  | |
| **% of AOP (preterm) cases requiring drug treatment** | *Extremely preterm* | 100% | 100% | 100% | 100% |
|  | *Very preterm* | 100% | 100% | 100% | 100% |
|  | *Moderate/late preterm* | 100% | 100% | 100% | 100% |
| **Non-AOP cases – provision of drug prophylaxis** | | | |  | |
| **% of non-AOP (preterm) cases requiring drug prophylaxis** | *Extremely preterm* | 100% | 100% | 100% | 100% |
|  | *Very preterm* | 100% | 100% | 100% | 100% |
|  | *Moderate/late preterm* | 100% | 100% | 0% | 100% |
| **At-facility provision constraint** | | | |  | |
| **% preterm cases requiring AOP drugs but not able to receive them (due to e.g. stock availability, bed/equipment capacity, HW capacity/skill)** | | 8.5% | | | |

**South Africa**

**National births**

| **Indicator** | | **Datapoint** |
| --- | --- | --- |
| **Total births (#k)** | *In 2021* | 1,166 |
|  | *In 2026* | 1,137 |
| **Annual growth in # births (2021-26)** | | -0.5% |
| **Sectoral split of birth location** | *Public* | 76% |
|  | *Private* | 20% |
|  | *Non-facility (home/community)* | 4% |
| **Split of births in public sector** | *Lower-level facilities** | 80% |
|  | *Higher-level facilities** | 20% |
| **Preterm/full term split of births** | *Preterm* | 21% |
|  | *Full term* | 79% |
| **Split of preterm births by gestational age sub-category** | *Extremely preterm (<28 wks)* | 7% |
|  | *Very preterm (28-32 wks)* | 8% |
|  | *Moderate/late preterm (32-37 wks)* | 84% |

**Referrals**

| **Indicator** | | **Datapoint** |
| --- | --- | --- |
| **Referrals – private sector to public sector:** | | |
| **% of preterm cases referred** | *Extremely preterm* | 10% |
|  | *Very preterm* | 5% |
|  | *Moderate/late preterm* | 0% |
| % attempted referrals unsuccessful** | | 20% |
| **Referrals – non-facility sector to public sector:** | | |
| **% of preterm cases referred** | *Extremely preterm* | 100% |
|  | *Very preterm* | 50% |
|  | *Moderate/late preterm* | 10% |
| % attempted referrals unsuccessful** | | 20% |
| **Referrals – lower-level public to higher-level public facilities:** | | |
| **% of preterm cases referred** | *Extremely preterm* | 100% |
|  | *Very preterm* | 20% |
|  | *Moderate/late preterm* | 0% |
| % attempted referrals unsuccessful** | | 20% |

**AOP Incidence**

| **Indicator** | | **Datapoint** |
| --- | --- | --- |
| **% preterm infants developing AOP** | *Extremely preterm* | 90% |
|  | *Very preterm* | 75% |
|  | *Moderate/ late preterm* | 20% |

**Treatment/prophylaxis provision assumptions (public sector facilities with ability to treat**

| **Indicator** | | **Base and Moderate cases** | | **High case** | |
| --- | --- | --- | --- | --- | --- |
|  |  | *2021* | *2026* | *2021* | *2026* |
| **AOP cases – provision of drug treatment** | | | |  | |
| **% of AOP (preterm) cases requiring drug treatment** | *Extremely preterm* | 100% | 100% | 100% | 100% |
|  | *Very preterm* | 100% | 100% | 100% | 100% |
|  | *Moderate/late preterm* | 100% | 100% | 100% | 100% |
| **Non-AOP cases – provision of drug prophylaxis** | | | |  | |
| **% of non-AOP (preterm) cases requiring drug prophylaxis** | *Extremely preterm* | 100% | 100% | 100% | 100% |
|  | *Very preterm* | 50% | 50% | 50% | 100% |
|  | *Moderate/late preterm* | 0% | 0% | 0% | 100% |
| **At-facility provision constraint** | | | |  | |
| **% preterm cases requiring AOP drugs but not able to receive them (due to e.g. stock availability, bed/equipment capacity, HW capacity/skill)** | | 20% | | | |
