# Supplementary material for: A landscape evaluation of caffeine citrate availability and use in newborn care across five low- and middle-income countries
Source: PLOS Glob Public Health. 2024 Jul 29;4(7):e0002486. doi: 10.1371/journal.pgph.0002486 (PMC11285928; doi:10.1371/journal.pgph.0002486)
Supplement: Attachment — Submitted filename: Response to Reviewers.docx [file pgph.0002486.s005.docx]

**Rebuttal Letter**

Dear Reviewer,

Thank you for your comments on our manuscript. Please see our responses to the comments you have raised.

Journal requirements

1.	The PLOS complete questionnaire on inclusivity in global research has been included and submitted as a “Supporting Information file” with the revised manuscript. It has also been appropriately referred to in the Methods section.

2.      Ethics statement:  this study included a survey of current practice asking clinicians’ opinions and practice allowing full “opt in” i.e. consent is implied by the fact the clinician responded to the survey. A formal ethics approval was not applied for.

3. The reference list has been reviewed. The reason for removing a referenced article is that we referenced it twice. Duplicated ones 7 and 28 have been deleted and corrected in the manuscript.

  Major comments:

The qualitative analysis involved 107 health care providers across 45 health care facilities. The spread of the providers into those in primary, secondary and tertiary health care facilities in addition to private hospitals gave a broad overview of the prescriptions and factors affecting the use of CC in the country. The distribution of health care providers interviewed has been provided in the methods. Country policies on the distribution of health care providers at different levels of care vary widely, so it was challenging to have the same types of providers interviewed at all levels of care in the 5 countries.

Additional information provided on the type of providers who answered the survey in the different countries has been provided in a new table 1.

Minor comments

The number of health care professionals interviewed is provided as 107 in the abstract on page 4. We have provided additional breakdown and analysis on the respondents in the methodology and discussion.

We have updated the SRA status for the missing products on table 4. The two blank products data was unavailable.

Updated methodology and forecast figure 2 with definition of sub-optimal treatment provided.

The statement “Based on the projected number of demand forecast, we estimated that out of 918.000 VLBW infants who met the AOP treatment received an appropriate referral for all countries” has been rephrased.

We have also updated the previous table 4 now table 5 - modelled out Unmet Need for Caffeine Citrate Treatment per country with percentages.

We have provided a supplement with additional information on the forecast.

Typographical errors in lines 44 and 45 have been corrected. Other typographical errors noticed have also been corrected.

Thank you once again for your comments on our manuscript.

Kind regards,

Olufunke Bolaji
