# Supplementary material for: A landscape evaluation of caffeine citrate availability and use in newborn care across five low- and middle-income countries
Source: PLOS Glob Public Health. 2024 Jul 29;4(7):e0002486. doi: 10.1371/journal.pgph.0002486 (PMC11285928; doi:10.1371/journal.pgph.0002486)
Supplement: Attachment — Submitted filename: Response to Reviewers PLOS Global Health.docx [file pgph.0002486.s006.docx]

**Response to Reviewers**

Thank you for reviewing our manuscript. Your comments are constructive and will improve the overall quality of the manuscript.

The greatest benefit of caffeine citrate in high income countries is seen in extremely preterm infants born below 28 weeks of gestational age. These infants do not survive in the LMIC setting and therefore the population of infants that would benefit from caffeine citrate in the LMIC is different: above 28 weeks of GA, very preterm and moderate preterm infants.

There is no clear evidence that caffeine will have the same effect on when applied in the LMIC setting. There are no published trials to show the effect of caffeine n the LMIC setting. It is
likely to have the positive effect but it would be essential to show that through the
research trial.

It is true that the greatest benefit of caffeine citrate would be to extremely preterm infants born below 28 weeks of gestational age. Even though the mortality of these infants is higher in low- and middle-income countries, there are several reports that show survival of these infants in those settings I have included some references of such studies here. Furthermore, the increasing use of antenatal corticosteroids for pregnant women at risk of preterm delivery as recommended by WHO has greatly improved the survival of these of extremely preterm infants are surviving in low resource settings.

- Survival of 24.3% for infants born at 24 to 27 weeks gestation in a tertiary centre in Nigeria (Fajolu, I., Eyanya Akintan, P., Ezenwa, B., & Chinyere Ezeaka, V. (2019). Survival of Extremely Preterm Neonates in a Resource-limited Setting. Iranian Journal of Neonatology, 10(3), 32-37. doi: 10.22038/ijn.2019.38772.1611)
- Survival of 53.6% in infants born at 26 weeks gestation (Niger J Paediatr 2020; 47 (2):61 – 67) DOI: http://dx.doi.org/10.4314/njp.v47i2.2
- Survival of 33% in infants born at less than 1000g in southwest Ethiopia (Bereka B, Demeke T, Fenta B, Dagnaw Y. Survival Status and Predictors of Mortality Among Preterm Neonates Admitted to Mizan Tepi University Teaching Hospital, Southwest Ethiopia. Pediatric Health Med Ther. 2021 Sep 1; 12:439-449. doi: 10.2147/PHMT.S319774. PMID: 34512074; PMCID: PMC8420788.)

2) It would be important to specify throughout the paper if authors are referring to intravenous or oral caffeine preparation. If there are available data on oral preparation of caffeine availability and pricing, it would be very important to include.
Oral caffeine citrate is much cheaper than the iv preparation and it is as effective. Giving oral caffeine will reduce the need for iv access, in particular central access through the umbilical venous catheter. This is very important to minimise the risk of severe infection and sepsis and it would greatly improve survival of these infants if sepsis can be avoided.
This paper should encourage the use of oral caffeine in the LMIC setting and the main positive effect of this paper would be to facilitate further research to prove the benefit of oral caffeine in the LMIC setting in very and moderately preterm infants above 28 weeks of gestation.

The overall aim of this study was to describe the demand, policies, and supply factors affecting the availability and clinical use of CC in LMIC and it was on the premise (as you have rightly stated) that there are no published trials to show the effect of caffeine in the LMIC setting, that this study was conducted. It is hoped that the knowledge shared from this paper will facilitate research including clinical trials on caffeine in LMIC settings.

The paper focused on the availability of caffeine citrate whether oral or intravenous knowing that oral caffeine was just as effective as intravenous caffeine. The data and findings reported are for both formulations. We have included the added advantage of the oral formulation for Caffeine Citrate in the discussion part of the manuscript.
